# The effect of implementing cognitive load theory-based design principles in virtual reality simulation training of surgical skills: a randomized controlled trial

**DOI:** 10.1186/s41077-016-0022-1

**Published:** 2016-06-07

**Authors:** Steven Arild Wuyts Andersen, Peter Trier Mikkelsen, Lars Konge, Per Cayé-Thomasen, Mads Sølvsten Sørensen

**Affiliations:** 1 Department of Otorhinolaryngology—Head and Neck Surgery, Rigshospitalet, Blegdamsvej 9, 2100 Copenhagen, Denmark; 2 Computer Graphics Lab, The Alexandra Institute, Aabogade 34, 8200 Aarhus, Denmark; 3 Copenhagen Academy for Medical Education and Simulation, Blegdamsvej 9, 2100 Copenhagen, The Capital Region of Denmark Denmark

**Keywords:** Cognitive load theory, Virtual reality simulation, Surgical skills training, Mastoidectomy, Temporal bone surgery

## Abstract

**Background:**

Cognitive overload can inhibit learning, and cognitive load theory-based instructional design principles can be used to optimize learning situations. This study aims to investigate the effect of implementing cognitive load theory-based design principles in virtual reality simulation training of mastoidectomy.

**Methods:**

Eighteen novice medical students received 1 h of self-directed virtual reality simulation training of the mastoidectomy procedure randomized for standard instructions (control) or cognitive load theory-based instructions with a worked example followed by a problem completion exercise (intervention). Participants then completed two post-training virtual procedures for assessment and comparison. Cognitive load during the post-training procedures was estimated by reaction time testing on an integrated secondary task. Final-product analysis by two blinded expert raters was used to assess the virtual mastoidectomy performances.

**Results:**

Participants in the intervention group had a significantly increased cognitive load during the post-training procedures compared with the control group (52 vs. 41 %, *p* = 0.02). This was also reflected in the final-product performance: the intervention group had a significantly lower final-product score than the control group (13.0 vs. 15.4, *p* < 0.005).

**Conclusions:**

Initial instruction using worked examples followed by a problem completion exercise did not reduce the cognitive load or improve the performance of the following procedures in novices. Increased cognitive load when part tasks needed to be integrated in the post-training procedures could be a possible explanation for this. Other instructional designs and methods are needed to lower the cognitive load and improve the performance in virtual reality surgical simulation training of novices.

## Background

Surgical procedures can be difficult to master for the novice regardless of training being based on virtual reality (VR) simulation, cadaveric dissection, or as supervised surgery in the operating room. Surgical procedural training often constitutes a complex learning task, requiring compound competencies within several domains, such as anatomical knowledge and technical skills. This can affect learning because of the limitations of cognitive processing. Cognitive load (CL) theory provides a useful framework for cognitive learning on the basis of the limitations in working memory [1], and CL theory is increasingly being considered in medical simulation [2].

According to the CL theory, three different components of information processing and learning contribute to the total CL experienced by the learner [3]: the *intrinsic* load of the learning task (inherent to the task itself), *extraneous* load of the learning situation (the way the task is presented), and the *germane* load of the learning process (the learning actually occurring). If these components combined exceed the cognitive capacities of the learner, the result could be a cognitive overload that inhibits the actual learning. Novices are especially at the risk of cognitive overload, and reducing the total cognitive load could therefore lead to a better performance and more efficient learning [1]. The components of cognitive load can be modified by several different strategies and design principles to provide better learning [3].

In VR surgical simulation, an example of a complex procedure that could benefit from optimizing the cognitive load is the mastoidectomy procedure in temporal bone surgery, which we have demonstrated induces a high cognitive load in novices [4]. The procedure requires navigation and handling of the virtual specimen, haptic drilling, and avoiding collisions with vital anatomical structures such as the dura, the facial nerve, the ossicles, and the inner ear including the semicircular canals. In addition, the learning condition involves different sources of information that need to be integrated (for example, visual information from the specimen and written instructions in which the appropriate information need to be sought out). This and other factors in the learning situation such as providing adequate problem solving methods and suitable guidance could place a substantial *extraneous* cognitive demand on the learner [3].

*Extraneous* load can be reduced by applying better approaches to the learning instructions and information or the learning tasks and goals [3]. We therefore hypothesized that the cognitive load of novices in VR simulation training of mastoidectomy could be reduced by implementing CL design principles to the simulation environment. In this study, we hypothesized that especially the extraneous load of the procedure could be lowered in the simulation environment and therefore applied two of the proposed task-related principles in the initial instructions of the procedure in order to reduce the extraneous load. There is currently limited knowledge on the relative effect sizes of the different CL-lowering strategies and we chose (1) the worked example principle, where a conventional task is replaced with the solution to be studied, and (2) the completion principle, where a conventional task is replaced with a partial solution the learner must finish [3]. We chose these strategies because these were feasible to implement in the studied VR simulator without extensive modifications to the simulation software, relevant in relation to reducing the extraneous load in the compound procedure studied, and based on theory could have a considerable effect on the extraneous load.

CL can be estimated by a range of different methods including subjective methods such as self-reported invested mental effort, stress level or perceived difficulty, and objective measures, including physiological or brain activity measurements and dual-task performance [5]. A reaction time test in the dual-task paradigm has been found to be a sensitive measurement of CL in surgical skills training of novices [6, 7], and we have previously applied this method to investigate cognitive load in VR simulation of mastoidectomy [4, 8]. Although this method is not able to discriminate between changes in the different components of CL, the method allows for repeated measurement and can be used to detect even minor changes in total cognitive load [8]. There are several general limitations in measuring cognitive load, and no particular measurement method is currently considered superior in estimating the cognitive load [9, 10].

In this study, we wanted to investigate the effect on CL of implementing worked examples and completion exercises in initial VR mastoidectomy training of novices using a secondary reaction time test for estimating CL. Secondly, we wanted to explore if the possible reduction of CL would be reflected in the following mastoidectomy performance.

## Methods

### VR simulation platform

**Fig. 1:**
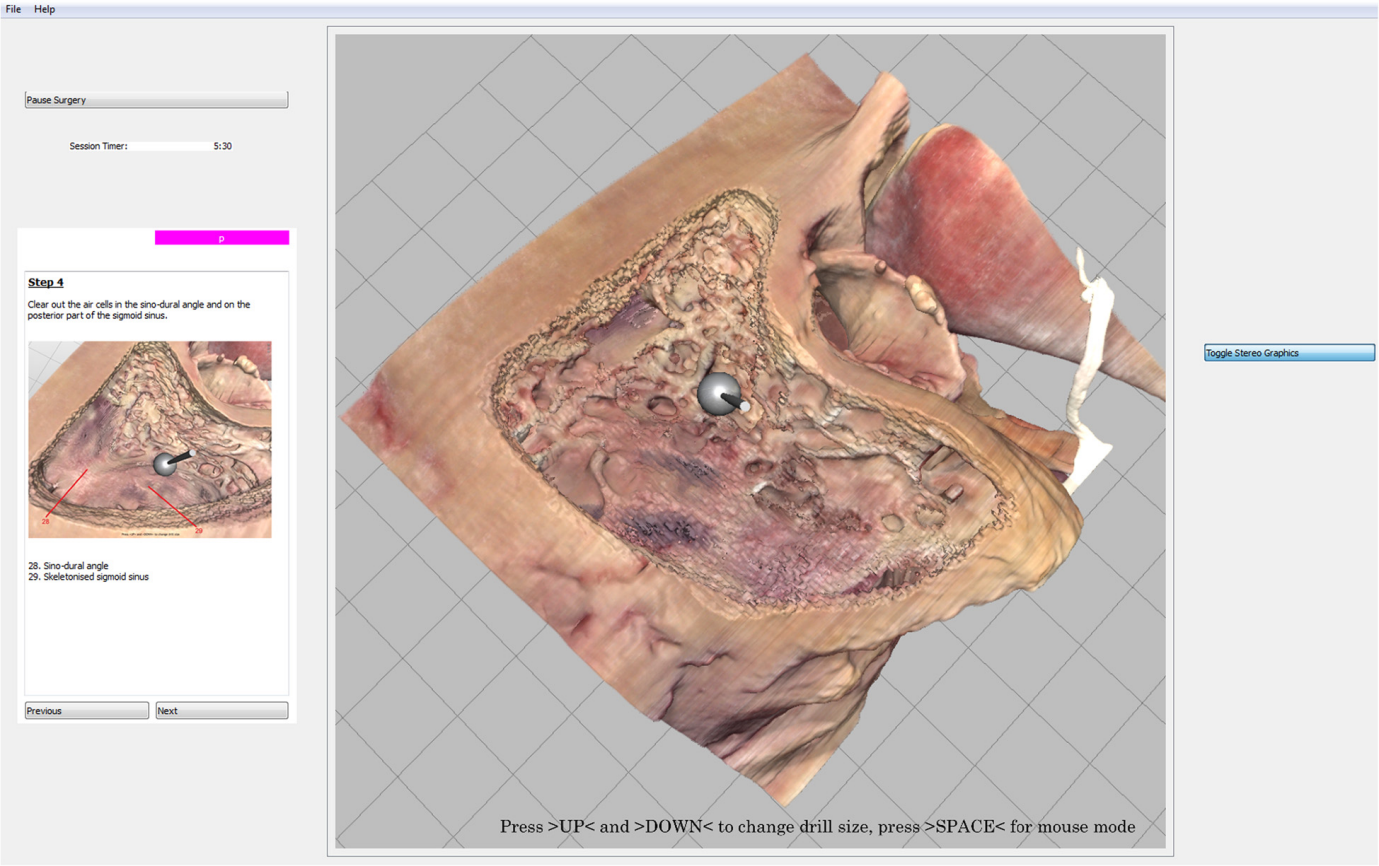
Screenshot from the Visible Ear Simulator with drilling of the virtual temporal bone. Unnecessary simulator controls are curtained through CThruView. The *purple box* above the (standard) instructions to the left illustrates the reaction time test

The Visible Ear Simulator is a VR temporal bone simulator based on cryo-sections of a human temporal bone [11, 12]. The simulation software is academic freeware available for download [13] and runs on a personal computer with a GeForce GTX® graphics card (Nvidia, USA). The Geomagic Touch® (3D Systems, USA) haptic device can be used for navigation and for drilling the virtual temporal bone with force feedback. A specially developed research version (version 1.3) of the simulator incorporated an integrated reaction time test in which participants were instructed to respond to a visual cue (color changing box) next to the on-screen instructions by pressing the keyboard corresponding to the letter displayed in the box (Fig. 1) [8]. Non-essential visual elements in the graphical user interface of the simulator were curtained using the freeware java application CThruView [14] (Fig. 1).

### Study-design, intervention, and randomization

**Fig. 2:**
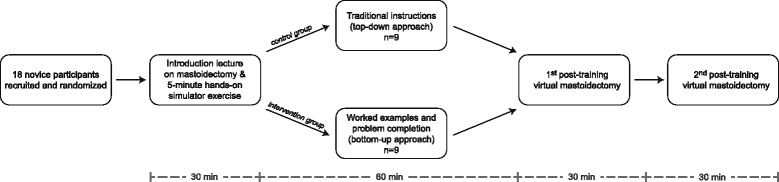
Flowchart and study design

The study was a randomized controlled trial of an educational intervention with a parallel design, a 1:1 allocation ratio, and blinding of the expert raters. Participants were randomized to the training intervention with sealed envelopes after having signed informed consent for participation and completing a background questionnaire. A flowchart is provided in Fig. 2.

All participants first received the same class lecture on the mastoidectomy procedure. This was followed by an individual 5-min hands-on simulator exercise unrelated to the procedure to familiarize participants with the haptic device and simulator navigation.

Participants in the control group received 1 h of self-directed training using the standard built-in simulator instructions with a step-by-step guide based on a top-down whole-task approach to the mastoidectomy procedure. In this traditional approach, the trainee works from the surface of the temporal bone towards the deep structures with no specific considerations to which parts of the procedure are of greatest difficulty.

The intervention group also received a 1-h self-directed training program. The training consisted of exploring worked examples with explanations emphasizing important aspects of the procedure. The worked examples were immediately followed by a corresponding problem completion exercise where participants had to drill a part task that combined the new key elements with previously practiced elements (segmentation with backward chaining part-task approach). The interventional program is detailed in the electronic supplemental material (Appendix). For both groups, all the instructions were presented on-screen within the simulator.

After the initial training, the participants performed two identical self-directed post-training procedures guided only by the standard on-screen instructional guide with 30 min allowed for each procedure.

### Sample size

Sample size calculation was based on cognitive load estimated by relative reaction time being the primary endpoint with data from a previous study on cognitive load during simulation training of the mastoidectomy procedure [8]: seven participants in each study arm would be sufficient to significantly detect a 10 % change in the relative reaction time between groups.

### Participants

Eighteen medical students from the Faculty of Health and Medical Sciences, University of Copenhagen, Denmark, volunteered for participation in this study, which was organized as an extracurricular activity. Participants from any semester were invited, and the only exclusion criterion was previous training in VR mastoidectomy. The study was conducted in October 2014 at the Simulation Centre at Rigshospitalet, Copenhagen, Denmark [15]. All the students were novices concerning the mastoidectomy procedure because it is not a part of the curriculum.

### Outcomes and statistics

The simulator measured the reaction time (in ms) several times at baseline (immediately before and after each of the two post-training procedures) in addition to three 60-s test rounds during the post-training procedures (resulting in >800 reaction time measurements in total). Data were analysed as previously [4, 8] with two times the SD used as a cutoff to define reaction time in the cases where participants missed the reaction time test or were too slow to react before the next reaction time test was presented. The reaction times were calculated relative to the individual baseline measurements (unitless) for each session to estimate individual changes in reaction time to enable comparisons between participants.

Final products of the two post-training sessions were auto-saved at 30 min and later assessed using a modified Welling scale for final-product analysis [16] by two blinded experts. Scores were calculated as the average score assigned by the two raters. The scale consists of 26 dichotomously rated items (1 point for adequate and 0 points for inadequate) totalling a maximum score of 26, which is the expected level of expert otosurgeons.

Data were analysed using linear mixed models for repeated measurements in IBM SPSS Statistics version 22 for MacOS X (IBM Corp., Armonk, NY, USA) with group, session, and for relative reaction time also tests round number, and interactions as main effects, and age and semester as covariates. The linear mixed models were iteratively improved to include only significant factors. *p* values below 0.05 were considered statistically significant.

## Results

All 18 participants completed the initial training and the two post-training sessions in the VR simulator and were included in the analysis. Baseline participant characteristics are provided in Table 1 (one questionnaire was incomplete). Participants in the intervention group were significantly older than the participants in the control group (25.9 vs. 22.9 years, *p* = 0.02). This was also reflected in the current semester of study (8.8 vs. 6.1) although this was not statistically significant (*p* < 0.10). The distribution of sexes was found to be equal in the two groups. No significant differences in the computer or gaming-related questions were found. Age and semester as covariates were not found to significantly contribute to the linear mixed models of either outcome and were excluded from the final model.

**Table 1:** Participant background and characteristics

Participant characteristic	Control group, mean	95 % CI	Intervention group, mean	95 % CI	*p*
Age (years)	22.9	21.3–24.4	25.9	23.5–28.2	0.02^*^
Sex					
Male (%)	33 %		25 %		0.73
Female (%)	67 %		75 %		
Semester	6.1	4.2–8.0	8.8	6.0–11.5	0.08
Average time on a computer/week (hours)	14.3	8.9–19.7	13.2	6.3–20.2	0.78
Self-rated computer skills (1–7, Likert like scale)	4.7	4.0–5.3	4.3	3.2–5.3	0.49
Current gaming frequency (1–5, Likert like scale)	2.3	1.1–3.6	1.7	0.8–2.5	0.33

*p* values calculated with analysis of variance (ANOVA)

^*^Significant at the <0.05 level

For the relative reaction time, the main effects of group (*p* = 0.02) and post-training session number (*p* < 0.01) contributed significantly to the model. The mean reaction time was found to be increased during the post-training procedures in both groups: the mean reaction time increased by 41 % in the control group and 52 % in the intervention group relative to individual baselines. Contrary to our hypothesis, the intervention group was thereby found to have an increased relative reaction time compared with the control group (Table 2).

**Table 2:** Relative reaction time (mean reaction time during simulation/mean reaction time during baseline)

	Control group	95 % CI	Intervention group	95 % CI	*p*
Post-training session 1	1.47	1.39–1.55	1.58	1.50–1.65	*p* = 0.01^*^
Post-training session 2	1.36	1.28–1.43	1.46	1.38–1.54	
Post-training mean	1.41	1.35–1.48	1.52	1.45–1.58	*p* = 0.02^*^

Estimated marginal means and *p* values calculated using linear mixed models

^*^Significant at the <0.05 level

For the final-product mastoidectomy performance, only the main effect of instructional group contributed significantly to the model (*p* < 0.005). The control group with standard instructions was found to outperform the intervention group by 2.4 points corresponding to a 19 % better performance (Table 3). For the first post-training session, the difference in performance between the two groups was even more pronounced (3.3 points). For the second post-training session, the performance of the intervention group increased and the difference in performance between the two groups became less distinct.

**Table 3:** Final-product performance of virtual mastoidectomy (26-item modified Welling scale, maximum score 26)

	Control group	95 % CI	Intervention group	95 % CI	*p*
Post-training session 1	15.5	13.6–17.4	12.2	10.2–14.1	0.45
Post-training session 2	15.4	13.9–16.9	13.8	12.1–15.6	
Post-training mean	15.4	14.4–16.5	13.0	11.8–14.4	<0.005^*^

Estimated marginal means and *p* values calculated using linear mixed models

^*^Significant at the <0.05 level

## Discussion

In this randomized controlled trial of the effect of implementing two CL-theory-based instructional design principles in the initial VR training of novices in the mastoidectomy procedure, we found that worked examples followed by a problem completion exercise did not reduce the reaction time in the secondary task nor the performance in the primary task in the post-training procedures. In contrast, the intervention increased the reaction time and reduced the subsequent performance of the procedure. In the following, we will discuss the change in reaction time as a change in CL as established in the literature [5, 6].

Few studies have investigated CL during surgical skills training. Adding mental arithmetic problems, and thereby additional CL, to surgical tasks in a VR laparoscopic simulator was found to increase primary task time to completion [17]. CL measured by mental effort questionnaires has been found to be correlated with time and number of movements in a salpingectomy/salpingotomy VR simulator [18]. This is the first study on the effect of implementing CL-theory-based design principles in initial instructions in a VR surgical simulator with post-training outcome measurements directly on the CL as well as on the primary task performance. A strength of our study is the randomized, controlled study design. The difference in age and semester between the two groups, resulting from the randomization, could not be shown to impact the CL or the final-product performance of the procedure most likely because the participants were all medical students with no exposure to temporal bone surgery, which is not a part of the curriculum. We have no reason to suspect differences in the participants’ cognitive capacity because of the similar background and the randomization, in addition to such measurement being difficult to meaningfully conduct. We found that the study size was sufficient to significantly demonstrate an effect of the intervention even though the effect was the opposite of what we hypothesized and the sample size therefore sufficient.

A limitation to our study is that we did not evaluate the CL and performance during the initial training session and therefore have no knowledge on whether the suggested principles reduced CL during the instruction. Nevertheless, an effect of reducing the CL during initial training should be durable in following training to be meaningful. In addition, any effect of repeated practice using the two different instructional approaches and the effect on the long-term retention of the procedure remains uninvestigated and should be addressed in future studies.

The differences in performance and relative reaction time between the intervention and control groups were most marked for the first post-training session, and by the second session, differences in reaction time and mastoidectomy performance between the intervention and control groups started to even out. The effect of repetition on final product and time to completion of the procedure is substantial especially in the initial part of the learning curve [19–21] and is likely to contribute to the performance increase from the first to the second procedure. Any positive longer term effect on learning of the intervention with the investigated CL-lowering design principles would have to exceed the effect of repetition alone, which both could be difficult to achieve and would require a dedicated study.

In our study, we found the CL to be increased for both the interventional group and control group, and we have previously found that CL is very high in traditional cadaveric dissection training of mastoidectomy as well [4]. This indicates that the mastoidectomy procedure places heavy cognitive demands on the surgical novice. Consequently, there is every reason to consider the CL in the learning situation. Cognitive overload detrimental to learning and skills acquisition can easily ensue in complex learning situations especially in the context of novice training. Some components of CL can be difficult to adjust: *germane load* is essential for learning and consists of the construction of cognitive schemas and should be optimized or increased rather than reduced and *intrinsic load* is dependent on the task and the level of element interactivity: by design, simulation often reduces element interactivity compared with more high-fidelity training modalities and this could be beneficial for low-expertise learners in the initial training [22]. However, *extraneous load* is non-essential for learning and often results from a poorly designed instruction which is the obvious area to improve first to manage CL in the learning situation.

In this study, we implemented the worked example and completion CL-theory-based design principles [3] into the simulation software. However, this also resulted in a part-task segmentation of the procedure during initial training. Part-task training of surgical technical skills has been much debated: a recent review of training design in relation to procedural skills in laparoscopic surgery concludes that empirical evidence of the efficacy of part-task training is scarce [23]. Conflicting results are found for simulation-based training of other procedures too: part-task training is reported to benefit skills acquisition and retention in central venous catheter education [24] whereas part-task training is not superior to a whole-task approach in flexible fibreoptic intubation training [25].

One reason for the inconsistent effect of part-task training of surgical technical skills could relate to the overall complexity of the procedures. Part-task approaches “work well if there are few interactions between the elements, but they do not work well if the elements are interrelated because the whole is then more than the sum of its parts” [26]. This could be a major explanation for the increased CL of the intervention group in our study. Even though our instructional intervention in the initial training utilized the part-task method of segmentation with backward chaining (prior task as successively added) [26] and not fractionation (deconstruction into individual elements trained separately), the integration of inter-related part tasks could contribute to rather than deal with complexity, adding to the germane load.

Worked examples are suggested to work by focusing the learner’s attention on only the necessary and relevant steps for the solution and completion tasks by presenting a partial solution to be completed, not needing to take all other potential steps into consideration [22]. Both principles could potentially reduce the *extraneous load* and reduce the total CL of the learning situation. Nevertheless, we find evidence of a negative effect on the following compound procedure, possibly because of increased cognitive resources being used on combining and integrating the inter-related part tasks. A balance should be found “between the advantages of whole-task practice and the disadvantages of cognitive overload caused by whole tasks that are too complex for learners” [26]. In terms of CL theory, part-task segmentation could increase the cognitive load used for the mental formation of schemas and connections and thereby increase the germane load to a point resulting in cognitive over load. This is especially a concern for novices with limited prior knowledge to anchor the part-task learning experiences to. In our study, we cannot separate the different components of cognitive load and establish that the intervention increased the germane load. However, it should in general be considered that there is some controversy on the issue of separating the different components of CL [7], and more validity evidence is needed for the different measures of CL including both secondary task performance and self-reported measures [9].

Most advanced VR surgical simulators are commercial and therefore difficult to modify to the necessary extent. However, in our study, we used a non-commercial simulator developed by the authors (PTM and MSS), making changes to the instructions and instructional design in relation to the studied CL lowering principles feasible. Other of the proposed design principles could be feasible in relation to other VR simulators, and the different CL lowering strategies should be considered in the specific context. Close collaboration between clinician experts, simulator developers, and medical educationalists could guide future VR simulator design because evidence-based training strategies are necessary to create training programs that allow novice trainees to learn complex but essential surgical procedures as efficiently as possible.

Simulation developers need to carefully reflect on the consequences of the instructional design choices, and our results indicate that VR simulation-based training of very complex psychomotor skills and procedures such as mastoidectomy should consider other CL-theory-based principles than the worked example and completion principles. These could for example be the integration of instructions and information directly into the visual operating field and by scaffolding instructions and feedback accommodating the novices’ needs. This should be explored in the future and could provide a better strategy for the novice learner than the CL-lowering strategies implemented in this study.

## Conclusions

In VR surgical simulation training of mastoidectomy, the standard instructions with a top-down approach to initial training of the procedure was found to provide less CL and a better performance compared with CL-theory-based training with instructions using worked examples followed by a problem completion task. Increased complexity of integrating the skills and knowledge gained with the part-task approach could be a possible explanation for the increased CL during the compound post-training procedures. In novice surgical technical skills training, it is likely that the overall complexity of the procedure such as mastoidectomy requires whole-task rather than part-task training and other CL-reducing approaches and strategies should be investigated to optimize the learning situation and reduce the cognitive load.

## Abbreviations

CL, cognitive load; VR, virtual reality

## Appendix

The interventional training program consisting of worked examples (WE) followed by problem completion (PC) exercises (part-task segmentation with backward chaining):

1. Chorda and facial nerve exposed and the triangle removed (WE).
2. Remove the triangular bony plate and avoid collisions with the facial nerve and chorda tympani (PC).
3. Identify stapes and the round window niche (WE).
4. Expose the chorda and facial nerve and remove the triangular bony plate (PC).
5. The thinned posterior ear canal wall and relations to the nerves (WE).
6. Thin the posterior ear canal wall, remove the remaining cells, and follow the nerves (PC).
7. The tip of the mastoid and the digastric ridge as a landmark (WE).
8. Exenterate the tip of the mastoid and identify the digastric ridge and the relation with the facial nerve (PC).
9. The middle ear: demonstration of incus, malleus, and the lateral semicircular canal as landmarks (WE).
10. Blueline the lateral semicircular canal (PC).
11. Exposure of the attic and identification of the incus and malleus (PC).
12. The antrum mastoideum (WE).
13. Drill to identify antrum mastoideum (PC).
14. The sigmoid sinus and relations (WE).
15. Remove the cells over sinus sigmoideus (PC).
16. Tegmen mastoideum and the dura (WE).
17. Remove the cells of tegmen mastoideum (PC).
18. Demonstration of the problem with overhang (WE).
19. Remove the overhang (PC).
20. The sino-dural angle (WE).
21. Remove the cells in the sino­dural angle and leave it sharp (PC).
22. The superficial borders of the mastoidectomy (WE).
23. Mark the superficial borders of the mastoidectomy (PC).
24. The saucerized cavity between the superficial borders (WE).
25. Saucerize the cavity and complete further steps as time permits (PC).

## References

[1] Sweller J (1988). Cognitive load during problem solving: effects on learning. Cogn Sci..

[2] Fraser KL, Ayres P, Sweller J (2015). Cognitive load theory for the design of medical simulations. Simul Healthc..

[3] van Merriënboer JJ, Sweller J (2010). Cognitive load theory in health professional education: design principles and strategies. Med Educ..

[4] Andersen SA, Mikkelsen PT, Konge L (2016). Cognitive load in mastoidectomy skills training: virtual reality simulation and traditional dissection compared. J Surg Educ.

[5] Brünken R, Plass JL, Leutner D (2003). Direct measurement of cognitive load in multimedia learning. Educational Psychologist..

[6] Rojas D, Haji F, Shewaga R (2014). The impact of secondary-task type on the sensitivity of reaction-time based measurement of cognitive load for novices learning surgical skills using simulation. Stud Health Technol Inform..

[7] Haji FA, Rojas D, Childs R, de Ribaupierre S, Dubrowski A (2015). Measuring cognitive load: performance, mental effort and simulation task complexity. Med Educ..

[8] Andersen SA, Konge L, Cayé-Thomasen P, Sørensen MS (2016). Cognitive load in distributed and massed practice in virtual reality mastoidectomy simulation. Laryngoscope..

[9] Naismith LM, Cavalcanti RB (2015). Validity of cognitive load measures in simulation-based training: a systematic review. Acad Med..

[10] Naismith LM, Cheung JJ, Ringsted C, Cavalcanti RB (2015). Limitations of subjective cognitive load measures in simulation-based procedural training. Med Educ..

[11] Sorensen MS, Mosegaard J, Trier P (2009). The visible ear simulator: a public PC application for GPU-accelerated haptic 3D simulation of ear surgery based on the visible ear data. Otol Neurotol..

[12] Trier P, Noe KO, Sorensen MS, Mosegaard J (2009). The visible ear surgery simulator. Stud Health Technol Inform..

[13] [The Visible Ear Simulator web site]. Available at: http://ves.cg.alexandra.dk. Accessed 4 Jan 2016.

[14] [CThruView Transparent Image Viewer web site]. Available at http://cthruview.sourceforge.net/. Accessed 4 Jan 2016.

[15] Konge L, Ringsted C, Bjerrum F (2015). The Simulation Centre at Rigshospitalet, Copenhagen, Denmark. J Surg Educ..

[16] Andersen SA, Cayé-Thomasen P, Sølvsten SM (2015). Mastoidectomy performance assessment of virtual simulation training using final-product analysis. Laryngoscope..

[17] Cao CG, Zhou M, Jones DB, Schwaitzberg SD (2007). Can surgeons think and operate with haptics at the same time?. J Gastrointest Surg..

[18] Bharathan R, Vali S, Setchell T (2013). Psychomotor skills and cognitive load training on a virtual reality laparoscopic simulator for tubal surgery is effective. Eur J Obstet Gynecol Reprod Biol..

[19] Andersen SA, Konge L, Cayé-Thomasen P, Sørensen MS (2015). Learning curves of virtual mastoidectomy in distributed and massed practice. JAMA Otolaryngol Head Neck Surg..

[20] Andersen SA, Konge L, Mikkelsen PT, Cayé-Thomasen P, Sørensen MS. Mapping the plateau of novices in virtual reality simulation training of mastoidectomy. Laryngoscope. 14 Apr 2016 [Epub ahead of print]. 10.1002/lary.26000 27075936

[21] Nash R, Sykes R, Majithi A (2012). Objective assessment of learning curves for the Voxel-Man TempoSurg temporal bone surgery computer simulator. J Laryngol Otol..

[22] van Merriënboer JJG, Kester L, Paas F (2006). Teaching complex rather than simple tasks: balancing intrinsic and germane load to enhance transfer of learning. Appl Cognit Psychol..

[23] Spruit EN, Band GP, Hamming JF, Ridderinkhof KR (2014). Optimal training design for procedural motor skills: a review and application to laparoscopic surgery. Psychol Res..

[24] Chan A, Singh S, Dubrowski A (2015). Part versus whole: a randomized trial of central venous catheterization education. Adv Health Sci Educ Theory Pract..

[25] Nilsson PM, Russell L, Ringsted C (2014). Simulation-based training in flexible fibreoptic intubation: a randomised study. Eur J Anaesthesiol..

[26] van Merriënboer JJG, Kester L. Whole-task models in education. In: Spector JM, Merrill MD, van Merriënboer JJG, Driscoll MP, eds. Handbook of research on educational communications and technology, 3rd ed. UK: Routledge; 2008. p. 441–456.

